# TGF-β Signaling in Cancer: Mechanisms of Progression and Therapeutic Targets

**DOI:** 10.3390/ijms26157326

**Published:** 2025-07-29

**Authors:** Elżbieta Cecerska-Heryć, Adrianna Jerzyk, Małgorzata Goszka, Aleksandra Polikowska, Julita Rachwalska, Natalia Serwin, Bartosz Wojciuk, Barbara Dołęgowska

**Affiliations:** 1 Department of Laboratory Medicine, Pomeranian Medical University of Szczecin, Powstancow Wielkopolskich 72, 70-111 Szczecin, Poland; 72464@student.pum.edu.pl (A.J.); malgosia@goszka.pl (M.G.); polikowska.aleksandra@gmail.com (A.P.); 73686@student.pum.edu.pl (J.R.); natalia.serwin@pum.edu.pl (N.S.); barbara.dolegowska@pum.edu.pl (B.D.); 2 Department of Immunological Diagnostics, Pomeranian Medical University of Szczecin, Powstancow Wielkopolskich 72, 70-111 Szczecin, Poland; bartosz.wojciuk@pum.edu.pl

**Keywords:** platelet growth factors, TGF-β, breast cancer, colorectal cancer, lung cancer

## Abstract

Transforming growth factor-β (TGF-β) is a key protein family member that includes activins, inhibins, and bone morphogenetic proteins (BMPs). It is essential in numerous biological processes, such as chemotaxis, apoptosis, differentiation, growth, and cell migration. TGF-β receptors initiate signaling through two primary pathways: the canonical pathway involving Smad proteins and non-canonical pathways that utilize alternative signaling mechanisms. When TGF-β signaling is disrupted, it has been shown to contribute to the development of various diseases, including cancer. Initially, TGF-β effectively inhibits the cell cycle and promotes apoptosis. However, its role can transition to facilitating tumor growth and metastasis as the disease progresses. Moreover, TGF-β drives cancer progression through epithelial–mesenchymal transition (EMT), modulation of factor expression, and evasion of immune responses. This complexity establishes the need for further research, particularly into pharmacological agents targeting TGF-β, which are emerging as promising therapeutic options. Current clinical and preclinical studies are making significant strides toward mitigating the adverse effects of TGF-β. This underscores the critical importance of understanding its underlying mechanisms to enhance treatment effectiveness and improve survival rates for cancer patients.

## 1. Introduction

It is impossible to overestimate the fundamental importance of biological signals in coordinating and conveying the activity of cells, tissues, and organs throughout an organism’s life. These signals regulate all facets of physiological development through membrane-bound proteins or released chemicals, including cell growth, differentiation, metabolism, and activity. Secreted hormones, cytokines, chemokines, and growth factors can interact locally or distantly with cell surface receptors. The family of signaling molecules known as transforming growth factor beta (TGF-β) has existed since the beginning of multicellular life. TGF-βs regulate cell differentiation, proliferation, and selectivity through various signaling pathways [1].

The uncontrolled growth of aberrant cells and the aberrant immune system’s recognition of these cells are characteristics of tumors [2]. Almost every tissue in the body can develop them, and each variety of cancer has unique characteristics. Tumorigenesis begins when a cell initiates its proliferation strategy and exceeds the normal bounds of cell division. Every cell that divides from this initial cell and its offspring exhibits aberrant proliferation [3].

The TGF-β signaling pathway plays a complex, two-phase role in cancer development. In the early stages, the compound exhibits suppressive impacts by inducing apoptosis and inhibiting the cell cycle, thus reducing cancer cell proliferation. However, as the disease progresses, cancer cells develop resistance to TGF-β signals. Instead of inhibiting tumor growth, TGF-β initiates cancer-promoting processes, including invasion, angiogenesis, and the formation of metastasis. This functional change highlights the adaptability of cancer cells and presents a significant difficulty in innovating effective therapies targeting this pathway [4].

## 2. Transforming Growth Factor β

### 2.1. Platelets

The first molecules to arrive at the site of tissue injury are platelets, which participate in the initial phases of inflammation and tissue repair. They provide a wide range of growth factors (GFs) produced from platelets to the areas of damage. Platelet-derived growth factors, such as PDGF (platelet-derived growth factor), VEGF (vascular endothelial growth factor), IGF-1 (insulin-like growth factor), HGF (hepatocyte growth factor), bFGF (basic fibroblast growth factor), TGF-β (transforming growth factor β), and EGF (epidermal growth factor), are among the GFs released by platelets [5]. Table 1 provides specifics on the function of growth factors.

Although platelets represent an essential source of TGF-β, they are not the only contributors. Other cell types also play a critical role in TGF-β production. Fibroblasts are major producers of TGF-β in the tumor stroma and in fibrotic tissues, where they contribute to extracellular matrix remodeling and immune modulation [14]. Macrophages, particularly tumor-associated macrophages (TAMs), secrete TGF-β as part of their immunosuppressive and tumor-promoting functions [15]. Activated T cells, especially regulatory T cells (Tregs), also release TGF-β, contributing to immune evasion in cancer [16]. Moreover, many cancer cells themselves are capable of producing TGF-β in an autocrine or paracrine manner, further amplifying pro-tumorigenic signaling in the tumor microenvironment [17].

All phases of tumor development, including tumor growth, tumor cell extravasation, and metastasis, include platelets as active players [18]. At the site of tissue damage, platelet activation triggers a series of events that promote tumor growth. Numerous proteins, including growth factors, lipids, and extracellular vesicles that contain genetic material, are released by platelets. These proteins can mediate the induction of phenotypic changes in target cells, including tumor, stromal, and immune cells, and encourage the development of cancer and metastases [19]. In the case of the platelet-secreted cytokine TGF-β, ligands from the TGF-β family can trigger signaling pathways in tumor cells that either prevent or promote carcinogenesis, depending on the stage of the disease and type of cancer. In both normal cells and the initial phases of tumor transformation carcinogenesis, TGF-β ligands activate signaling pathways. These pathways trigger the production of genes that prevent proliferation, promote cell differentiation, induce autophagy or apoptosis, suppress inflammation, and inhibit angiogenesis. On the other hand, TGF-β can also encourage the development of metastases in advanced cancer by regulating the extracellular matrix and microenvironment, triggering the production of chemokines, suppressing the immune system, and participating in the epithelial–mesenchymal transition (EMT) [20]. The role of growth factors is presented in Table 1.

### 2.2. TGF-β Family

The TGF-β superfamily comprises numerous regulatory proteins with similar biological functions and structural similarities. Activins and inhibins, Muller’s inhibitory substance, left–right determinant, lymph node growth differentiation factor, TGF-β, bone morphogenetic proteins (BMPs), growth and differentiation factors, and others are among its families [21]. TGF-β1, TGF-β2, and TGF-β3 are the three isoforms that currently make up the TGF-β family. The genes that encode each of the human isoforms are located on distinct chromosomes: TGF-β1 is encoded in the long arm of chromosome 19 (19q13.1), TGF-β2 is encoded in the long arm of chromosome 1 (1q41), and TGF-β3 is encoded in the long arm of chromosome 14 (14q24) [22]. Endothelial cells, hematological cells, and connective tissue all express TGF-β1, whereas neuronal and epithelial cells express TGF-β2, and mesenchymal cells mainly express the TGF-β3 gene [21].

### 2.3. TGF-β Signaling

#### 2.3.1. Formation and Secretion of Mature TGF-β

**Figure 1:**
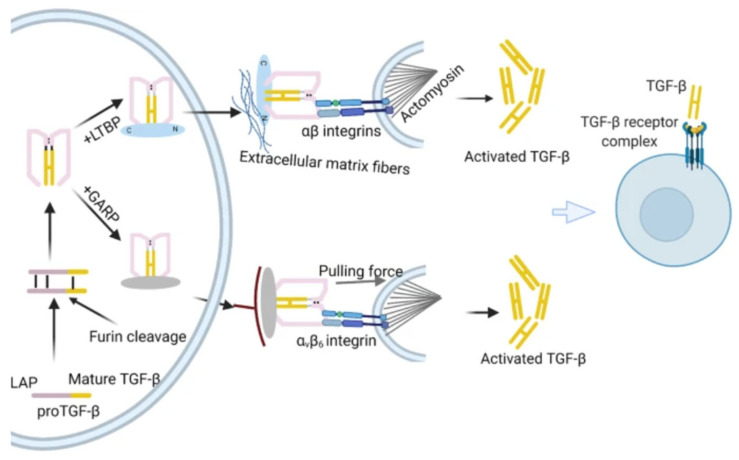
Formation of mature TGF-β [23].

The precursor molecules of TGF-β family proteins comprise a mature polypeptide, a prodomain (a peptide attached to the latency-associated protein LAP), and a signal peptide. The signal peptide removal is followed by proteolytic cleavage of the precursor by furin-type proteases. A mature TGF-β dimer, produced by the Golgi apparatus, forms a small latent complex (SLC) with latency-associated protein (LAP) through a non-covalent bond. After that, the SLC forms a large latent complex (LLC) with the latent TGF-β binding protein (LTBP). Thrombospondin 1 (TSP-1), glycoprotein A repeat dominant protein (GARP), integrins, and other TGF-β binding proteins are among the proteins and enzymes that activate latent TGF-β, transforming it into disulfide-linked dimers and homodimeric ligands. Via the LTBP, SLC is attached to extracellular matrix (ECM) proteins such as fibrillin and fibronectin. To attach TGF-β for integrin-induced activation, both LTBP and GARP are directly involved. Latent TGF-β becomes active and attaches to the TβR complex to control transcription with the aid of αβ integrins and mechanical force [23]. The pro-TGF-β activation is depicted in Figure 1.

#### 2.3.2. SMAD-Dependent TGF-β Signaling

**Figure 2:**
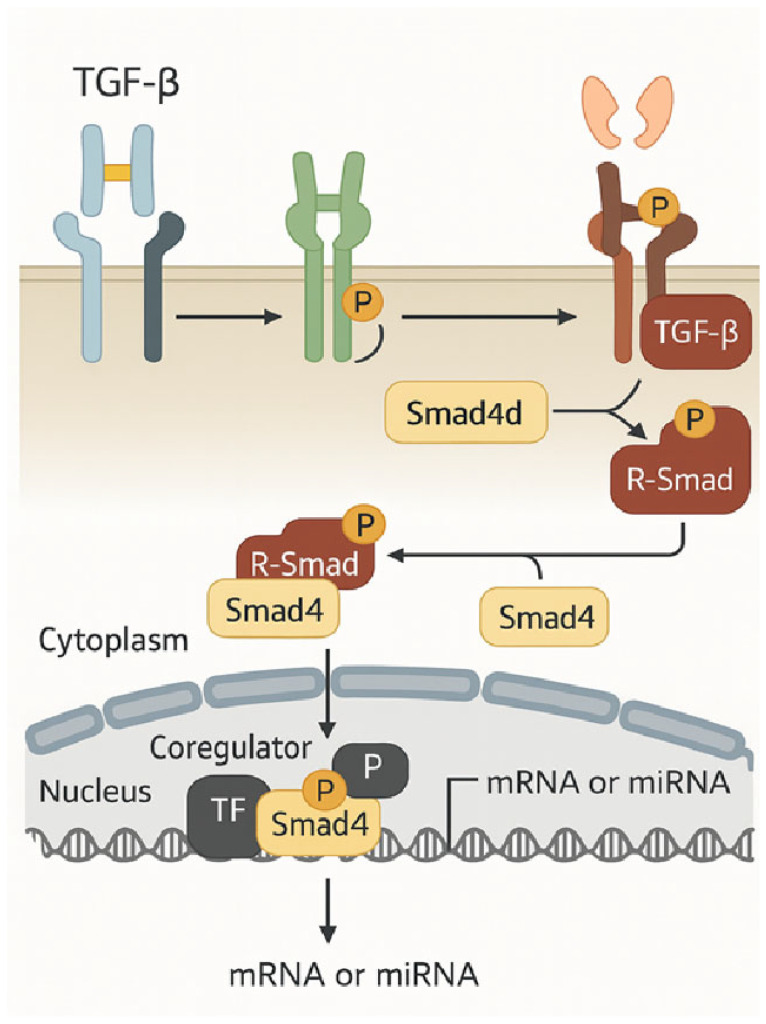
A simplified model of TGF-β-induced R-Smad activation leads to Smad-mediated gene expression activation [25].

Based on their comparable sequences, all families of secreted TGF-β and TGF-β-related factors bind and activate heteromeric receptor complexes on the cell surface, which are categorized into type I and type II receptors. Both receptor types are classified as dual-specificity kinases, possessing a cytoplasmic kinase domain that exhibits both tyrosine and serine/threonine kinase activity. The two type I receptors and the two type II receptors form heteromeric complexes with dimeric ligands, which allow the constitutively active kinases of the type II receptors to phosphorylate the periplasmic regions of the cytoplasmic domains of the type I receptors, thereby activating the type I receptor kinase [24]. Following their dissociation from the receptors, these “receptor-activated Smads” (R-Smads) join forces with Smad4 to form complexes that go to the nucleus, where they work in tandem with transcription factors and high-affinity DNA-binding coregulators to either activate or repress individual genes. R-Smad is recruited and activated in conjunction with clathrin-dependent endocytosis. Also crucial are inhibitory Smads (Smad6 and Smad7), which block functional Smad activation by binding to type I receptors and either preventing R-Smad activation or inhibiting the formation of the R-Smad complex with Smad4 [25]. Figure 2 illustrates a simplified model of TGF-β-induced R-Smad activation, which leads to Smad-mediated stimulation of gene expression.

**Figure 3:**
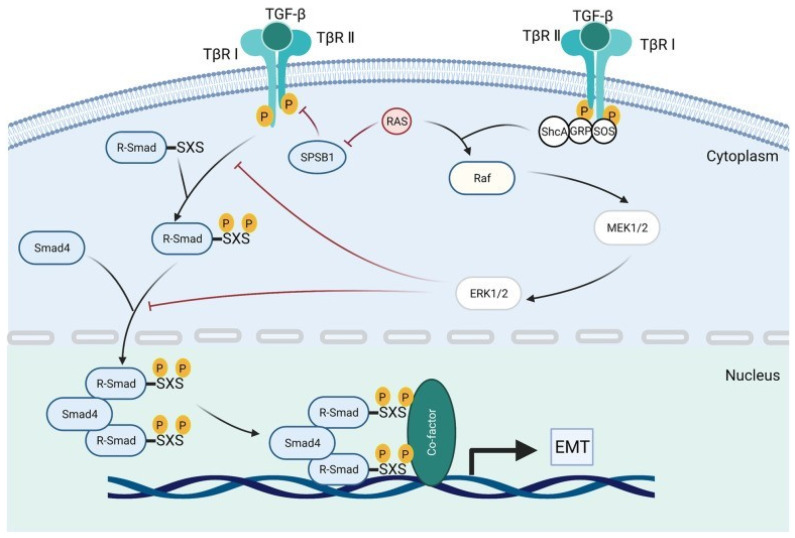
Activation of SMAD signaling by TGF-β in cancer.

In cancer, the TGF-β canonical pathway plays a dual role. Initially, it acts as a tumor suppressor, but in later stages, it can promote tumor progression and metastasis. TGF-β binds to a receptor complex on the cell membrane, first to TβRII and subsequently to TβRI. The activated TβRI receptor initiates a signaling cascade by phosphorylating the SMAD2 and SMAD3 proteins. These proteins then form a complex with SMAD4. The resulting SMAD2/3/4 complex translocates to the nucleus of the cell, where it regulates the expression of target genes involved in processes such as epithelial–mesenchymal transition (EMT), cell cycle control, and apoptosis [26]. Figure 3 illustrates the activation of SMAD signaling by TGF-β in tumors.

#### 2.3.3. SMAD-Independent TGF-β Signaling

Non-Smad signaling pathways are the aggregate term for all routes and downstream cascades that TGF-β triggers via phosphorylation, acetylation, sumoylation, ubiquitination, and “protein–protein” interactions. These interactions, commonly referred to as non-Smad signaling pathways, mediate the intracellular responses to TGF-β or associated molecules. The pathways of phosphatidylinositol 3-phosphatidyl kinase (PI3K)/AKT, c-Jun amine-terminal kinase (JNK), mitogen-activated protein kinase (MAPK), extracellular signal-regulated kinase 1/2 (Erk1/2), Rho-like signaling, and kinase p38 mitogen-activated protein kinase (p38/MAPK are all activated by mature TGF-β [23]. Besides Smad, these receptor-activated transducers may mediate signaling responses independently or in conjunction with Smad, convergently regulating Smad activity [25].

**Figure 4:**
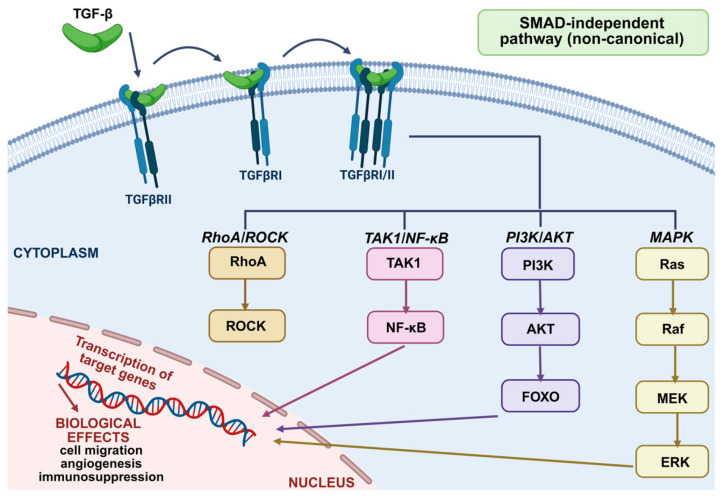
Activation of non-SMAD signaling by TGF-β in cancer [27].

The activation of the non-canonical pathway by TGF-β plays a crucial role in tumor progression by regulating processes such as cell migration, angiogenesis, immunosuppression, and treatment resistance. When TGF-β binds to the TGFBR1/TGFBR2 receptor complex, it can activate signaling pathways independently of SMAD proteins. This activation occurs through various pathways, including the MAPKs (ERK), PI3K/AKT, Rho-like GTPases, TAK1/NF-κB, and ROCK kinase pathways. These alternative pathways influence gene expression and the inflammatory response by promoting epithelial–mesenchymal transition (EMT), thereby creating a tumor microenvironment that supports growth and metastasis [17]. Figure 4 illustrates the activation of non-SMAD signaling by TGF-β in tumors.

Non-canonical pathways also influence processes that promote cancer progression. In pancreatic cancer, non-canonical TGF-β signaling, mediated through TAK1 and p38, increases tumor cell resistance to chemotherapy and promotes stromal fibrosis within the tumor. In breast cancer, the activation of PI3K/AKT and JNK pathways facilitates epithelial-to-mesenchymal transition (EMT), resulting in a loss of cell adhesion and an increased potential for metastasis [28]. In gliomas and non-small cell lung cancer, the TGF-β pathway is associated with MAPK activation and increased PD-L1 expression, which enhances immunosuppression and contributes to resistance to immunotherapy [29].

Understanding the role of non-canonical TGF-β signaling pathways is essential for developing effective cancer therapies. This is especially important in cancers that have significant immunosuppressive and fibrotic characteristics, such as pancreatic cancer, non-small cell lung cancer (NSCLC), and gliomas, where simply blocking Smad pathways is not enough [30].

## 3. Autophagy–EMT Interplay

Autophagy is a lysosome-dependent degradation process that breaks down cytoplasmic components, including macromolecules and organelles such as mitochondria and the endoplasmic reticulum. The most prevalent form, macroautophagy, proceeds through stages of initiation, nucleation, elongation, maturation, fusion, and degradation [31].

In cancer biology, autophagy plays a dual role. It can act as a tumor suppressor by removing damaged proteins and organelles, thereby maintaining cellular homeostasis. Loss of the *BECN1* gene, which encodes Beclin 1, essential for phagophore formation, has been observed in breast, prostate, ovarian, hepatocellular, and cervical squamous-cell carcinomas. Its deletion reduces autophagy and enhances tumor cell proliferation, while knockdown of other autophagy genes also inhibits tumor growth [32].

Conversely, in advanced tumors, autophagy promotes survival under stress conditions, such as hypoxia and nutrient deprivation. Autophagy-deficient cells show impaired viability, and gene knockdown experiments confirm that autophagy inhibition can induce tumor cell death [33].

TGF-β plays a crucial role in epithelial–mesenchymal transition (EMT), a reversible and dynamic biological process in which epithelial cells lose their cell–cell adhesion and apical–basal polarity, acquiring mesenchymal properties such as motility, invasiveness, and resistance to apoptosis. TGF-β is considered one of the most potent inducers of EMT in various cell types, including carcinoma cells [34]. The process involves both Smad-dependent and Smad-independent signaling pathways, leading to the activation of EMT transcription factors, such as SNAIL, SLUG, ZEB1/2, and TWIST, which repress epithelial markers, like E-cadherin, and upregulate mesenchymal markers, like vimentin and N-cadherin [35].

EMT plays a fundamental role in cancer progression, especially in promoting invasion, metastasis, and resistance to therapy [36]. It is increasingly recognized that EMT is not a binary switch but rather a spectrum of intermediate states, often referred to as “partial EMT,” which confer plasticity and adaptability to tumor cells [37]. This dynamic transition also contributes to immune evasion and the development of drug resistance. Notably, the interaction between autophagy and EMT is emerging as a critical aspect of tumor adaptation. On one hand, autophagy supports EMT by supplying energy and biosynthetic precursors necessary for cytoskeletal remodeling and cellular motility during metastasis. It also prevents the accumulation of damaged organelles and protein aggregates generated during EMT-associated stress [38].

On the other hand, autophagy may suppress EMT in early tumor development by degrading key transcription factors that promote EMT, thereby maintaining epithelial identity. Additionally, TGF-β itself can induce autophagy through interconnected signaling pathways, suggesting the existence of a tightly regulated feedback loop [39].

Understanding the crosstalk between TGF-β signaling, autophagy, and EMT provides essential insights into the molecular basis of tumor progression and offers promising therapeutic targets for cancer treatment. Pharmacological modulation of autophagy or inhibition of specific EMT regulators may represent effective strategies for treating invasive and treatment-resistant cancers [38,40,41].

## 4. Cancers

Cancer remains the second leading cause of death worldwide, with approximately 9.6 million fatalities reported in 2018, accounting for one in six deaths. Its global impact continues to grow due to aging populations and changes in environmental and lifestyle risk factors. Men are more frequently diagnosed with lung, prostate, colorectal, stomach, and liver cancers, while women more commonly develop breast, colorectal, lung, cervical, and thyroid cancers.

The majority of cancer-related deaths result from malignant tumors, which are characterized by uncontrolled cell proliferation, the ability to invade surrounding tissues, and the potential to metastasize to distant organs. These features are recognized as hallmarks of cancer [42].

Cancer is also marked by the evasion of immune surveillance and sustained proliferative signaling—features that have led to its description as a “wound that never heals” [2]. Early detection remains critical in the fight against cancer. Unlike normal cells, which respond appropriately to regulatory signals, cancer cells grow and divide uncontrollably, damaging tissues and organs, and eventually spreading throughout the body if left unchecked [43].

## 5. Lung Cancer

Lung cancer is the second most common cancer worldwide, predominantly affecting individuals aged 65 and older, with an average diagnosis age of 70 [44]. Early detection through screening and symptom monitoring significantly improves outcomes [45]. Cigarette smoking is the leading preventable risk factor, alongside chronic infections, asbestos, radon, air pollution, and genetic or molecular alterations [46,47]. Understanding these factors supports the development of more effective prevention strategies [47].

**Figure 5:**
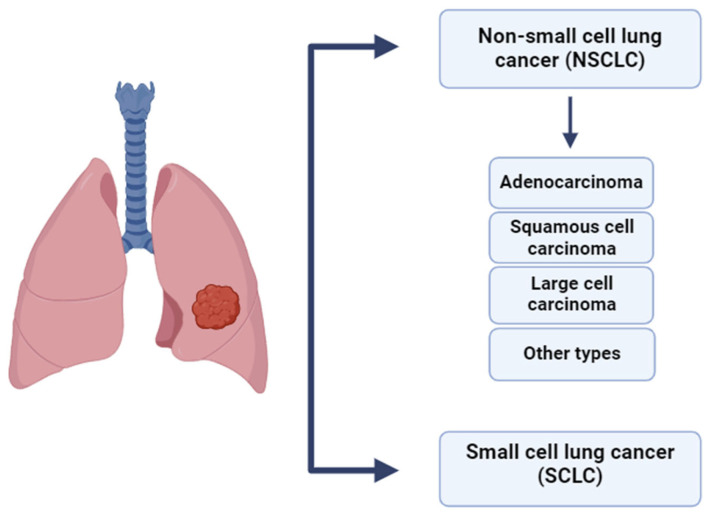
Histological classification of lung cancer [47,48].

Lung cancer is classified into small cell lung cancer (SCLC) and non-small cell lung cancer (NSCLC), with NSCLC comprising 80–85% of cases. Its main subtypes include adenocarcinoma (~40%), squamous cell carcinoma (25–30%), and large cell carcinoma (10–15%) [47]. Advances in immunophenotyping have facilitated more accurate classification of poorly differentiated tumors, thereby reducing the incidence of large cell carcinoma diagnoses [48]. The histological classification of lung cancer is presented in Figure 5.

### 5.1. The Multifaceted Role of TGF-β in Lung Cancer Progression

#### 5.1.1. Introduction: TGF-β as a Central Regulator in Lung Cancer

**Figure 6:**
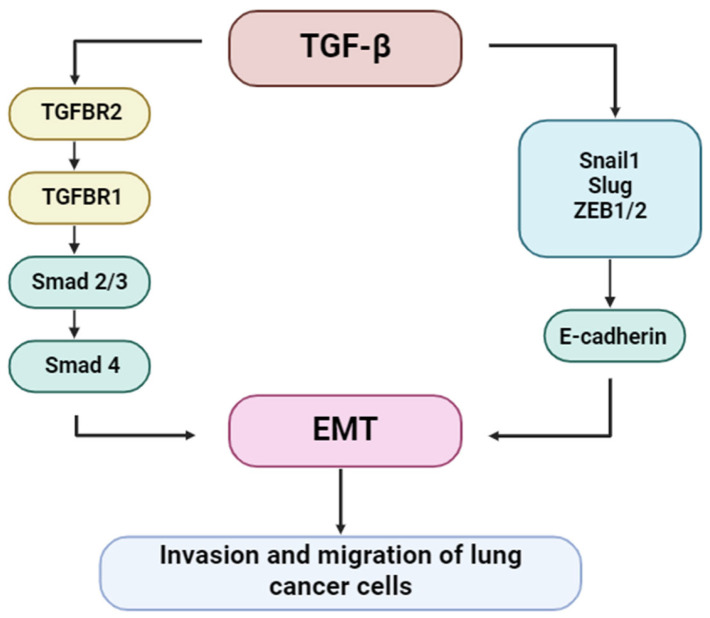
Regulation of lung cancer cell invasion and migration by TGF-β signaling.

Lung cancer (LC) remains one of the deadliest malignancies worldwide, with poor long-term survival despite therapeutic advancements. Among the molecular pathways implicated in LC progression, transforming growth factor β (TGF-β) has emerged as a central regulator of tumor biology. TGF-β orchestrates a wide spectrum of oncogenic processes, including immune evasion, epithelial-to-mesenchymal transition (EMT), metastasis, and remodeling of the tumor microenvironment. Figure 6 illustrates the role of TGF-β signaling in regulating lung cancer cell invasion and migration [49].

#### 5.1.2. Immune Evasion Through NK Cell Suppression

TGF-β plays a pivotal role in impairing the function of natural killer (NK) cells, a critical component of the innate immune system responsible for identifying and eliminating tumor cells. By downregulating NKG2D ligands (NKG2DLs) on the surface of lung cancer cells, TGF-β effectively reduces NK cell recognition and cytotoxicity. Experimental evidence from studies in A549, NCI-H23, and SW-900 lung cancer cell lines indicates that TGF-β reduces the expression of ULBP1-3 and MICA/B in a cell-type-specific manner. These immunoevasive effects can be reversed pharmacologically using galunisertib, an ALK5 inhibitor. Furthermore, TGF-β enhances the expression of matrix metalloproteinases (MMP2, MMP9, MMP14), which may contribute to the proteolytic shedding of NKG2DLs, further promoting immune escape [50].

#### 5.1.3. Epithelial-to-Mesenchymal Transition (EMT)

**Figure 7:**
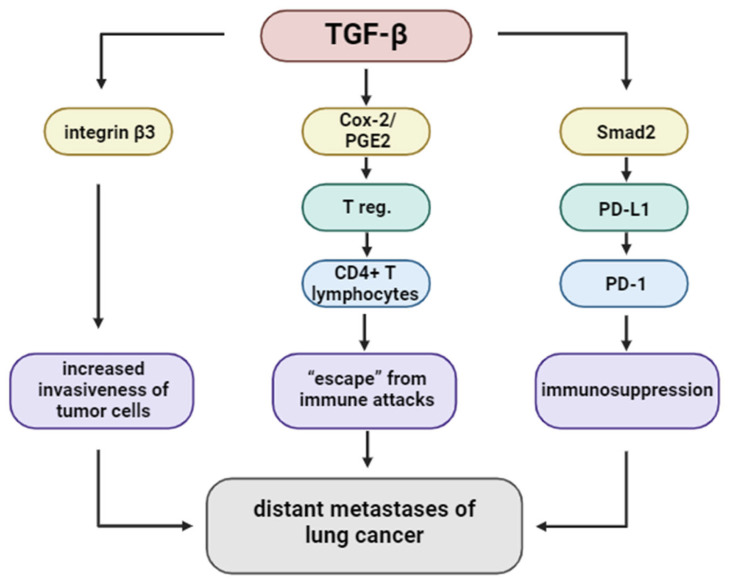
The TGF-β signaling pathway regulates the distant metastasis of lung cancer.

TGF-β is a potent inducer of EMT, a biological process through which epithelial cells acquire mesenchymal characteristics, including enhanced motility and invasiveness. This transition is critical for tumor dissemination and metastasis. TGF-β triggers EMT primarily through the canonical Smad2/3 signaling axis, which induces EMT-associated transcription factors such as Snail, Slug, and ZEB1/2. These repress epithelial markers, such as E-cadherin, and promote the expression of mesenchymal proteins, including vimentin and N-cadherin. Non-canonical signaling pathways also contribute to the regulation of EMT. Figure 7 depicts the molecular relationship between TGF-β signaling and EMT progression in lung cancer [49].

#### 5.1.4. Regulation by Non-Coding RNAs

Non-coding RNAs, including microRNAs (miRNAs) and long non-coding RNAs (lncRNAs), significantly influence the TGF-β signaling cascade. Oncogenic miRNAs such as miR-3614-5p, miR-128-3p, and miR-9 promote EMT by targeting TGF-β inhibitors or enhancing EMT transcription factors. In contrast, tumor-suppressive miRNAs, such as miR-132 and miR-422a, inhibit TGF-β-induced EMT by targeting pathway components, including TGF-β1, SMAD2, and associated EMT markers. These regulatory mechanisms highlight the importance of miRNA-mediated fine-tuning of TGF-β signaling in lung cancer progression [50,51].

#### 5.1.5. Endothelial Transmigration and Metastatic Dissemination

TGF-β facilitates metastatic spread by modulating tumor–endothelial cell interactions. It enhances integrin β3 expression, which promotes adhesion of tumor cells to endothelial cells, particularly in the lymphatic system. This interaction is crucial for transendothelial migration, enabling cancer cells to infiltrate the circulation. Furthermore, TGF-β influences Notch signaling and stimulates the formation of invadopodia, enabling matrix degradation and facilitating passage through endothelial barriers. These findings underscore the multifactorial role of TGF-β in promoting lung cancer metastasis [52,53,54].

#### 5.1.6. Induction of Regulatory T Cells (Tregs) via COX-2/PGE2 Axis

TGF-β fosters immunosuppression not only by affecting innate immunity but also by promoting the expansion of regulatory T cells (Tregs). It induces COX-2 expression and PGE2 production in CD4+ T cells, leading to the differentiation of FOXP3+ regulatory T cells (Tregs). The co-expression of TGF-β, COX-2, and FOXP3 has been detected in NSCLC tissues, indicating an active immunosuppressive microenvironment. Inhibition of either COX-2 or PGE2 pathways has been shown to reduce Treg generation and restore effector T cell function, suggesting potential synergy with immunotherapeutic strategies [50,55,56,57,58].

#### 5.1.7. Immune Checkpoint Modulation

TGF-β contributes to immune evasion by upregulating immune checkpoint molecules, especially PD-L1. In A549 lung adenocarcinoma cells, TGF-β1 induces PD-L1 expression via Smad2-dependent transcriptional activation of the CD274 gene. Notably, Smad3 does not replicate this effect, suggesting specificity in the downstream signaling pathway. Additionally, non-canonical pathways involving myocardin-related transcription factor A (MRTF-A) and NF-κB/p65 have been shown to enhance PD-L1 expression. PLK1-mediated phosphorylation of vimentin facilitates Smad2 nuclear translocation, further supporting this mechanism. Together, these pathways demonstrate how TGF-β signaling intersects with immune checkpoint regulation to suppress anti-tumor immunity [58,59,60].

#### 5.1.8. Tumor–Stroma Interactions

Within the tumor microenvironment, TGF-β activates cancer-associated fibroblasts (CAFs), which secrete extracellular matrix components and soluble factors that support tumor growth and invasion. TGF-β also promotes angiogenesis by stimulating endothelial cells and enhancing vascular endothelial growth factor (VEGF) production. These stromal interactions contribute to a supportive niche for tumor expansion. Clinical observations suggest that high stromal TGF-β activity is associated with a poor prognosis in lung adenocarcinoma, underscoring its relevance as a therapeutic target [57].

#### 5.1.9. Prognostic and Predictive Relevance of TGF-β

TGF-β serves as a powerful prognostic and predictive biomarker in lung cancer. Elevated levels of TGF-β in extracellular vesicles are correlated with resistance to immune checkpoint inhibitors, shorter progression-free survival, and poorer overall survival. Furthermore, TGF-β-inducible genes such as TGFBI, along with specific miRNAs and lncRNAs, have been identified as potential diagnostic and prognostic indicators. These molecular signatures, summarized in Table 2, offer promising tools for patient stratification and therapeutic decision-making [50,52].

In conclusion, TGF-β signaling is a central mediator of lung cancer progression, influencing a wide range of tumor-intrinsic and extrinsic processes. From EMT induction and immune evasion to stromal remodeling and metastatic dissemination, TGF-β drives multiple aspects of tumor biology. Targeting the TGF-β pathway—either directly or in combination with immunotherapy—represents a promising strategy to overcome therapeutic resistance and improve outcomes for patients with lung cancer. Continued exploration of TGF-β signaling will be vital for developing precision medicine approaches in thoracic oncology.

## 6. Colorectal Cancer

With a 9.2% global death rate, colorectal cancer (CRC) ranks fourth in terms of cancer-related causes of death and is the second most frequent disease in women and the third most common in men. Men have a 25% higher risk of morbidity and death than women, and their 5- and 10-year survival rates are 65% and 58%, respectively [61].

Regardless of age or race, males are approximately 1.5 times more likely than women to get colorectal cancer. On the other hand, tumors of the right side of the colon, which have a more aggressive course, are more common in women. It is essential to comprehend these gender disparities in risk to prevent and treat cancer effectively. The risk of this disease is directly increased by several factors, including smoking, inflammatory bowel disease, alcoholism, diabetes, genetic mutations, trans fats, cholesterol, saturated fatty acids, carbohydrates, proteins, and a so-called “pro-inflammatory” diet, among many others [62].

### 6.1. TGF-β in Colorectal Cancer

#### 6.1.1. The Dual Role of TGF-β Signaling in Colorectal Cancer Progression

Transforming growth factor β (TGF-β) exhibits a dual and context-dependent role in colorectal cancer (CRC). In the healthy intestine, it maintains immune tolerance and epithelial integrity by suppressing the activation of immune cells and promoting barrier function. However, in advanced tumors, TGF-β shifts toward a pro-tumorigenic function—an effect referred to as the *TGF-β paradox*. Disruption of TGF-β signaling leads to chronic inflammation, epithelial transformation, and tumor–stroma crosstalk that promotes cancer progression [63].

Approximately 15% of CRCs are classified as microsatellite instability-high (MSI-H) and carry inactivating mutations in the TGFBR2 gene, particularly within a 10-adenine repeat tract. These mutations impair type II receptor function, leading to the loss of canonical TGF-β signaling [63]. In contrast, microsatellite-stable (MSS) tumors often exhibit chromosomal instability (CIN) and loss of heterozygosity (LOH) affecting various components of the TGF-β pathway. Notably, the restoration of TGFBR2 in MSI-H CRC cells alters β1-integrin glycosylation, thereby impacting adhesion and migration, and thus linking TGF-β signaling to cell-surface remodeling [64].

Additionally, TGF-β-induced glycosylation changes contribute to tumor progression by affecting the activity of other oncogenic pathways, such as the Notch pathway, further amplifying the phenotypic plasticity of CRC cells [63].

Germline mutations in mismatch repair (MMR) genes, such as MLH1, MSH2, MSH6, and PMS2, lead to the loss of MMR protein expression in dMMR tumors. The 10-adenine repeat tract in the coding region of TGFBR2 is one of the most common mutational hotspots in dMMR/MSI-H CRC. Preclinical studies in mouse models have demonstrated that loss-of-function mutations in TGFBR2 may promote the transformation of normal colonic epithelial cells into malignant ones. Moreover, the disruption of TGFBR2 signaling, in combination with chronic colonic inflammation, can drive invasive colorectal tumorigenesis, in part through the recruitment and activation of tumor-associated macrophages (TAMs) [63].

#### 6.1.2. EMT, Metastasis, and the Emerging Role of the USF2/S100A8 Axis in TGF-β Signaling

TGF-β is a well-established inducer of epithelial–mesenchymal transition (EMT), a critical process that enables tumor cells to acquire invasive and metastatic capacities. Recent studies have expanded our understanding of this mechanism by identifying the transcription factor USF2 and the calcium-binding protein S100A8 as key downstream mediators of TGF-β-driven EMT in colorectal cancer (CRC) [62,64].

Functional experiments have demonstrated that TGF-β stimulation enhances USF2 expression, which subsequently activates *S100A8* transcription. This molecular axis promotes EMT by upregulating mesenchymal markers such as vimentin and Snail, while concurrently downregulating epithelial markers like E-cadherin, thereby facilitating cellular plasticity and motility [62,63]. Morphological analysis of CRC cell lines further confirmed that high S100A8 expression correlates with spindle-like phenotypes and increased invasiveness. In contrast, its low expression is associated with epithelial morphology and reduced metastatic potential [62].

Importantly, immunohistochemical analysis of 412 CRC tissue samples revealed that cytoplasmic co-expression of USF2 and S100A8 strongly associates with advanced TNM stage, lymph node involvement, distant metastases, and shorter overall survival, suggesting their clinical relevance as prognostic markers [65]. Interestingly, extracellular S100A8 was found to negatively regulate this axis, suggesting a potential autocrine feedback mechanism that modulates the strength of TGF-β signaling in the tumor microenvironment [65].

In addition to this transcriptional regulation, recent findings also suggest a link between TGF-β receptor signaling and remodeling of cell-surface glycosylation. Restoration of TGFBR2 expression in MSI-H CRC cells alters β1-integrin glycosylation patterns, which can significantly affect cell–matrix adhesion and migration, hallmarks of metastatic progression [65]. These post-translational modifications provide an additional, non-genetic layer of control through which TGF-β signaling may influence tumor invasiveness.

#### 6.1.3. Prognostic Relevance of TGF-β Pathway Components

Multiplex immunohistochemistry performed on CRC tissue microarrays revealed increased expression of TGF-β1, TGFBRI, TGFBRII, SMAD4, SMAD2/3, SMAD1/5/9, and phosphorylated SMAD2/3 in tumor tissue compared to adjacent mucosa [66,67]. Among these, high expression of TGFBRII and p-SMAD1/5/9 was associated with improved overall survival. Reduced expression of SMAD4 and p-SMAD2/3 correlated with worse prognosis. Genomic analysis based on The Cancer Genome Atlas (TCGA) further confirmed that SMAD3, SMAD4, and TGF-β1 are positively associated with survival, whereas SMAD2, SMAD6, SMAD7, SMAD9, TGFBRI, and TGFBRII are negatively associated [65,66].

These data underline the prognostic potential of TGF-β signaling components and support their use as predictive biomarkers and therapeutic targets in CRC.

Taken together, these studies highlight a broader network of TGF-β-driven pro-metastatic pathways in CRC. The USF2/S100A8 axis, in particular, appears to mediate a crucial link between TGF-β signaling and EMT, while glycosylation-related modulation of adhesion further supports tumor cell dissemination. These mechanisms not only enhance our understanding of CRC progression but also open new therapeutic avenues targeting EMT-regulatory nodes within the TGF-β pathway.

The role of TGF-β signaling in colorectal cancer is complex and highly context-dependent. While physiologically essential for maintaining intestinal homeostasis, its dysregulation—whether through genetic mutations such as TGFBR2 loss in MSI-H tumors, epigenetic alterations, or microenvironmental factors—contributes significantly to CRC progression, EMT, and metastasis. Emerging data highlight the importance of non-canonical mediators such as the USF2/S100A8 axis and post-translational modifications, including altered glycosylation, in amplifying the pro-metastatic effects of TGF-β. In parallel, multiplex analyses and genomic profiling have revealed that expression levels of TGF-β pathway components (e.g., SMADs, TGFBRI/II, S100A8) are closely associated with clinical stage, survival, and tumor aggressiveness. Understanding the exact mechanics of the TGF-β signaling pathway in CRC is vital and has enormous promise. It could result in more potent treatments for this kind of cancer. There is promise in the fight against colorectal cancer since therapeutic approaches that target the cytokine’s signaling may offer a novel treatment option. However, further investigation is required to identify people who could benefit from such treatments.

Table 3 presents the information listed in this chapter on the factors and processes that, through the TGF-β signaling pathway, influence the progression or inhibition of colorectal cancer

## 7. Breast Cancer

Breast cancer (BC) is the most commonly diagnosed malignancy and remains the leading cause of cancer-related mortality among women worldwide. In 2022, approximately 2.3 million women were diagnosed globally, and around 670,000 deaths were attributed to the disease [67]. This high global burden highlights the ongoing need for intensified research, early detection efforts, and effective public health strategies.

According to the American Cancer Society (ACS), approximately 310,720 new cases of invasive breast cancer and 56,500 cases of ductal carcinoma in situ (DCIS) are expected to be diagnosed in U.S. women in 2024, alongside an estimated 42,250 deaths [68]. DCIS represents a non-invasive form confined to the milk ducts, whereas invasive breast cancer involves the infiltration of surrounding tissue and the potential for distant metastasis.

Most breast cancer cases are diagnosed in women aged 55 and older, with an average age at diagnosis of around 62. Fewer than 10% of cases occur in women under 45, and while rare, breast cancer can also affect men, accounting for about 1% of cases in the U.S. [69]. Female sex and increasing age are major risk factors. Additionally, 5–10% of BC cases are hereditary, primarily associated with BRCA1 and BRCA2 gene mutations [70,71].

Breast cancer is generally classified into non-invasive and invasive stages. Non-invasive forms such as DCIS and lobular carcinoma in situ (LCIS) are confined to ducts or lobules and may progress if untreated [72,73]. Invasive breast cancer is characterized by the penetration of cancer cells beyond the basement membrane into adjacent tissues, with the potential to spread to the liver, lungs, brain, and bones [74].

The most common invasive subtypes include the following:

- Invasive ductal carcinoma (IDC)—the predominant subtype.
- Invasive lobular carcinoma (ILC)—diffuse growth within lobules.
- Medullary carcinoma—less common, with clear tumor boundaries.
- Mucinous carcinoma—composed of mucus-producing cells, typically with a favorable prognosis.
- Inflammatory breast cancer—aggressive, with skin erythema and edema.
- Paget’s disease of the nipple—affects the nipple and areola.
- Tubular carcinoma—a well-differentiated form with excellent prognosis [75,76].

**Figure 8:**
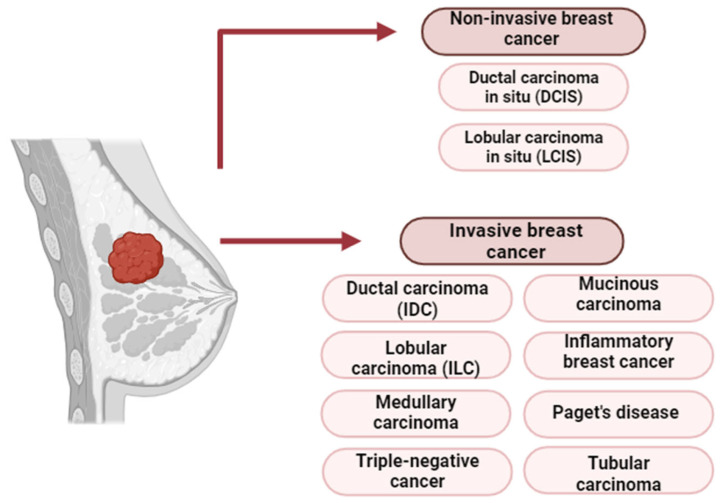
Histological classification of breast cancer [78].

Triple-negative breast cancer (TNBC) is a clinically aggressive subtype of IDC, lacking estrogen (ER), progesterone (PR), and HER2 receptor expression. TNBC accounts for 10–15% of breast cancer cases and is more prevalent in younger women, especially those carrying BRCA1 mutations. These mutations are strongly linked to early-onset disease and TNBC phenotypes [77]. The histological subtypes of BC are summarized in Figure 8. 

### 7.1. The Role of TGF-β Signaling in Breast Cancer Progression

#### 7.1.1. Breast Cancer and the Significance of TGF-β

Breast cancer is among the most prevalent malignancies worldwide, alongside colorectal and lung cancer. Understanding its molecular mechanisms is essential for improving diagnosis, management, and treatment outcomes. One such mechanism is the transforming growth factor-β (TGF-β) signaling pathway, which plays a pivotal role in breast cancer progression [23].

#### 7.1.2. TGF-β Signaling Pathways in Cancer

TGF-β signaling influences key cellular processes, including proliferation, differentiation, apoptosis, immune evasion, epithelial–mesenchymal transition (EMT), and metastasis. It operates through both canonical (Smad-dependent) and non-canonical pathways, including Rho-like GTPase, PI3K/AKT, and Ras-Erk cascades. Notably, TGF-β directly phosphorylates ShcA, leading to Ras activation and downstream signaling via c-Raf, MEK, and Erk, all of which contribute to tumor progression [23].

#### 7.1.3. ΔNp63α and the FBXO3 Axis in TGF-β-Induced EMT

The p63 protein, a member of the p53 family, exists in isoforms such as TAp63 and ΔNp63. Although rarely mutated in cancer, ΔNp63α has a significant influence on metastasis, cell survival, and EMT regulation. ΔNp63α controls expression of EMT-related genes like Twist1, ZEB1, and E-cadherin and is itself regulated by E3 ubiquitin ligases such as FBXO3 [79].

Niu et al. demonstrated that TGF-β1 stabilizes FBXO3, which facilitates the degradation of ΔNp63α, enhancing EMT and metastatic potential via non-canonical pathways [79]. In MCF-10A mammary epithelial cells, TGF-β1 reduced ΔNp63α levels, activated Smad3, and altered EMT markers. Restoration of ΔNp63α reversed these effects and suppressed TβRI-induced migration and invasion [79]. Importantly, inhibition of canonical Smad signaling did not prevent ΔNp63α downregulation, but MEK inhibition did—indicating that this process is Erk-dependent and Smad-independent. TGF-β selectively increased FBXO3 expression, confirming its role in ΔNp63α degradation and metastasis promotion [23].

#### 7.1.4. Crosstalk Between TGF-β and EGFR Pathways

TGF-β’s role contrasts with that of epidermal growth factor (EGF), which is primarily mitogenic. However, both signaling pathways interact and can synergistically promote EMT, invasion, and metastasis [80]. Dysregulation of EGFR is common in cancers such as breast, lung, and colon cancer.

In a study using 67 breast cancer tissue samples, immunohistochemistry revealed that 59.7% of the samples were positive for TGF-β, and 37.3% were positive for EGFR. Co-expression of both markers was found in 29.9% of cases. Their co-expression correlated with worse prognosis and reduced overall survival [80].

#### 7.1.5. NETs and Chemoresistance in the Tumor Microenvironment

Metastasis remains the leading cause of breast cancer mortality. The tumor microenvironment, particularly components such as cytokines, growth factors, and immune cells, plays a crucial role in treatment resistance. Inflammatory cells such as neutrophils form neutrophil extracellular traps (NETs), which are now recognized as contributors to tumor progression and chemoresistance [79,80,81,82,83].

Mousset et al. found that chemotherapy-exposed cancer cells released IL-1β, which induced NET formation. NETs activated latent TGF-β through integrin αvβ1 and MMP9. TGF-β, in turn, induced EMT, promoting resistance and survival in metastatic BC cells [84]. This IL-1β–NET–TGF-β axis highlights the interplay between the immune system and tumor environment in chemoresistance [85].

#### 7.1.6. TGF-β–Integrin–SOX4 Axis in Immunotherapy Resistance

Triple-negative breast cancer (TNBC) is aggressive and often resistant to treatment. Tumor-infiltrating lymphocytes (TILs) improve prognosis in TNBC, but immune evasion mechanisms persist. A CRISPR screen identified ITGAV (encoding integrin αv) and SOX4 as mediators of resistance to cytotoxic T lymphocytes [81].

SOX4, a transcription factor induced by TGF-β, is overexpressed in many cancers. Integrin αvβ6 activates latent TGF-β, which upregulates SOX4, thereby promoting resistance. Inactivation of ITGAV reduced SOX4 levels. Blocking αvβ6 with monoclonal antibodies decreased SOX4 and TGF-β signaling, sensitizing TNBC cells to CD8+ T cells. Conversely, treatment with active TGF-β reversed this effect [81]. These findings suggest that the integrin αvβ6–TGF-β–SOX4 axis contributes to immune escape and poor outcomes [79,81]. This axis may offer novel opportunities for targeted therapy in breast cancer [79].

#### 7.1.7. Summary and Clinical Implications

Table 4 summarizes the key molecular components involved in TGF-β signaling and their roles in breast cancer progression.

TGF-β signaling significantly contributes to breast cancer progression by modulating EMT, immune escape, and chemoresistance. Its interaction with EGFR, ΔNp63α, NETs, and integrins illustrates a complex network of oncogenic regulation. Therapeutic targeting of the TGF-β pathway—particularly through inhibitors of integrins or downstream effectors like SOX4—offers promising potential for improving outcomes in aggressive breast cancer subtypes such as TNBC.

Ongoing research into cytokine inhibitors and TGF-β blockade may lead to personalized treatment strategies, offering hope for more effective therapies and improved prognosis.

The transforming growth factor-β (TGF-β) signaling pathway plays a multifaceted and context-dependent role in the progression of breast cancer. It promotes epithelial–mesenchymal transition (EMT), enhances metastatic potential, and contributes to resistance mechanisms by modulating the tumor microenvironment and immune escape pathways. Key molecular players such as ΔNp63α, FBXO3, EGFR, neutrophil extracellular traps (NETs), and SOX4 illustrate how TGF-β signaling interconnects with oncogenic processes, including chemoresistance and immunotherapy failure.

Notably, the synergistic activity between the TGF-β and EGFR signaling pathways, the FBXO3-mediated degradation of metastasis suppressors, and the integrin αvβ6–TGF-β–SOX4 axis represent potential therapeutic targets, especially in aggressive subtypes such as triple-negative breast cancer (TNBC). The ability of TGF-β to both drive tumor progression and modulate immune responses underscores its value not only as a prognostic biomarker but also as a promising candidate for future precision therapies.

Advancing our understanding of TGF-β-related mechanisms will be crucial for developing novel strategies to overcome treatment resistance and improve long-term outcomes in breast cancer patients.

## 8. Challenges and Potential of TGF-β Inhibitors in Cancer Therapy

TGF-β signaling is a challenging target due to its dual role and pleiotropic nature, suggesting that patient selection and therapeutic dosing of TGF-β medications must be carefully managed [86].

Soluble receptors that target TGF-β and operate as ligand traps or neutralizing antibodies have demonstrated anticancer and antifibrotic properties in several preclinical investigations. Therefore, employing ligand traps or neutralizing antibodies to block the TGF-β ligand’s access to receptors is a viable approach in cancer treatment. The most researched medication in this class of neutralizing antibodies is GC1008 (fresolimumab), a human monoclonal antibody specifically engineered to neutralize all three types of TGF-β [87].

Additionally, research has demonstrated that 264RAD, a human monoclonal antibody targeting the αvβ6 integrin, can inhibit tumor development and metastasis in vivo. Patients with high-risk and trastuzumab-resistant breast cancer may benefit from targeting αvβ6 with 264RAD antibody alone or in conjunction with trastuzumab, an antibody that neutralizes HER2 [86,88].

Two vaccines, Vigil (Gemogenovatucel-T) and Lucanix (Belagenpumatucel-L), have been developed in conjunction with TGF-β antisense. The first vaccine to enter a Phase III study is Lucanix, a non-viral, gene-based allogeneic tumor cell vaccine targeting TGF-β2. Lucanix’s impact on patients with non-small cell lung cancer (NSCLC) was examined in Phase III due to its favorable safety profile and improved survival compared to Phase II. Nevertheless, the study’s survival target was not met; the median survival was 20.3 months for the vaccine group versus 17.8 months for the placebo, indicating no significant difference. Experts suggest that combined treatment with immunotherapy, radiation, or chemotherapy may enhance survival, underlining the need for further research [88].

The extracellular domains of TβRII and betaglycan have been fused with immunoglobulin fragment crystallizable (Fc) to create soluble sTβRII-Fc and sBetaglycan-Fc, respectively. Targeting TGF-β using these decoy receptors has shown tumor and metastasis inhibition in preclinical models. Muraoka et al. demonstrated that sTβRII-Fc reduced lung metastases, tumor cell motility, and intravasation, and induced apoptosis in primary tumors. Similarly, sBetaglycan-Fc prevented lung metastases and tumor growth in MDA-MB-231 xenografts [89,90].

A critical recent development involves small-molecule TGF-β receptor kinase inhibitors, notably galunisertib (LY2157299). Galunisertib is a selective ATP-mimetic inhibitor of TGFBR1 (ALK5) and is the most clinically advanced agent in this class. Preclinical studies have demonstrated that galunisertib inhibits both canonical (SMAD-dependent) and non-canonical (MAPK, PI3K/AKT) TGF-β signaling, reduces EMT, and reverses TGF-β-mediated immune suppression in vitro and in vivo [91]. Clinically, galunisertib has been tested in Phase I/II trials. In an NSCLC cohort receiving galunisertib (150 mg b.i.d., 14 days on/14 days off) plus nivolumab (3 mg/kg q2w), the overall response rate was 24%, and the median progression-free survival was 5.26 months. Median OS was ~12 months [92]. Preclinical and early-phase data in hepatocellular carcinoma similarly showed acceptable safety and signals of efficacy [93].

Other kinase inhibitors, such as vactosertib (TEW-7197) and SB-431542, also target ALK5 and exhibit similar anti-TGFβ effects in early-stage studies [86].

Inhibiting TGF-β expression and activation, blocking ligand–receptor interactions, or inhibiting receptor kinase signaling are the three primary approaches for targeting TGF-β. Cancer research continues to focus on these strategies through clinical evaluation of neutralizing antibodies, vaccines, antisense oligonucleotides, and small-molecule inhibitors. Preclinical evidence supports that TGF-β pathway inhibition can overcome immunosuppression, reduce tumor progression, fibrosis, EMT, and angiogenesis—a multipronged anticancer strategy [94].

A promising therapeutic strategy is to combine TGF-β pathway inhibitors with current cancer treatments, such as immunotherapy (for example, PD-1/PD-L1 inhibitors), chemotherapy, or radiotherapy [95].

The PD-1/PD-L1 pathway allows tumors to evade the immune response by inhibiting the activity of cytotoxic T lymphocytes and natural killer (NK) cells. Blocking this pathway can restore anti-tumor immunity; however, the effectiveness of this treatment can sometimes be diminished by the secondary expression of PD-L1 in the tumor microenvironment (TME). Simultaneously inhibiting the TGF-β and PD-L1 pathways may offer greater benefits than targeting each pathway individually, as these pathways function independently within the TME. Bintrafusp alfa, a bifunctional molecule that combines a PD-L1 inhibitor with a TGF-β trap, demonstrates more potent anti-tumor activity in vivo compared to using two separate drugs. This enhanced effectiveness is due to its ability to act simultaneously and locally within the TME [93].

TGF-β levels increase following chemotherapy, which promotes epithelial–mesenchymal transition (EMT), the development of cancer stem-like cells (CSCs), and drug resistance, particularly in triple-negative breast cancer (TNBC). In preclinical models of TNBC, a combination of a TGF-β inhibitor (LY2157299) and paclitaxel effectively halted the expansion of the CSC population and prevented relapse after treatment with paclitaxel alone. In the examined mouse tumors, this combination therapy resulted in a lower relapse rate compared to chemotherapy alone [96]. Examples of therapies targeting the TGF-β pathway include antibodies, vaccines, and kinase inhibitors, as listed in Table 5.

## 9. Summary and Conclusions

Transforming growth factor β (TGF-β) has a crucial but context-dependent role in cancer biology. In early tumorigenesis, it acts as a tumor suppressor by inhibiting proliferation and inducing apoptosis. In contrast, in advanced cancers, TGF-β promotes disease progression through processes such as epithelial–mesenchymal transition (EMT), metastasis, immune evasion, and remodeling of the tumor microenvironment.

Research indicates that TGF-β signaling contributes to tumor plasticity and immune resistance by suppressing natural killer (NK) cells and upregulating matrix metalloproteinases (MMPs). These immunosuppressive effects can be counteracted by pharmacological inhibitors, such as galunisertib, which enhance the effectiveness of immune responses in lung cancer.

Additionally, TGF-β signaling is regulated by non-coding RNAs, including microRNAs and long non-coding RNAs, which affect EMT, metastasis, and drug resistance in various cancers. Elevated TGF-β levels are associated with poor responsiveness to immunotherapies and shorter survival, particularly in lung and breast cancer.

Therapeutic targeting of the TGF-β pathway requires careful patient selection and is often combined with other treatments, such as immune checkpoint inhibitors. Further research into the interactions between TGF-β signaling and immune and stromal networks is crucial for the development of personalized therapies.

In summary, targeting the TGF-β signaling axis presents significant potential for enhancing cancer treatment outcomes, especially in aggressive and treatment-resistant tumors, with future studies focusing on biomarker-guided approaches.

## Figures and Tables

**Table 1:** The role of growth factors.

Growth Factor	Role
EGF	It controls cell survival, proliferation, migration, and differentiation contributes significantly to wound healing and tissue homeostasis [6].
PDGF	It promotes the production of glycosaminoglycans and collagens I and III aids in wound healing. Additionally, it lowers platelet aggregation via regulating vascular tone and taking a role in angiogenesis [7].
VEGF	It is essential for disease-related angiogenesis and contributes to vascular expansion and the formation of lymphatic vessels [8]. It enhances the permeability of existing blood vessels and promotes the development of new ones [9].
IGF-1	It reduces blood glucose levels while blocking insulin secretion, binds to insulin receptors, promotes glucose transport in muscle and fat, and prevents the liver from producing glucose [10].
HGF	In various organs, it promotes the growth, migration, morphogenesis, and angiogenesis of epithelial cells. It helps injured livers, kidneys, and lungs heal on their own. Additionally, it uses anti-inflammatory and anti-apoptotic signals to protect both epithelium and non-epithelial organs [11].
bFGF	It holds therapeutic promise for wound healing and in the treatment of cardiac, cerebrovascular, and neurological systemic disorders. It is implicated in the proliferation and differentiation of several mesoderm—and neuroectoderm-derived cell types [12].
TGF-β	In the early phases of tumor development, it suppresses tumor growth and has an antiproliferative impact. On the other hand, it promotes tumor growth in later stages by facilitating the autocrine loop of TGF-β, which aids in the spread of metastases [13].

**Table 2:** Significance of transforming growth factor β (TGF-β) in lung cancer progression.

Factor/Process	Description	Source
NKG2DL	TGF-β causes ↑ an increase in the expression of NKG2DL, an activating receptor for NK cells. This situation causes ↓ a decrease in the activation of NK cells, resulting in ↓ a reduction in the immune response against tumor cells.	[49]
MMP	The induction of MMPs by TGF-β promotes the metastasis and invasion of tumor cells.	[49]
EMT	TGF-β causes ↑ an increase in the expression of EMT-inducing transcription factors. These factors cause ↓ the expression of E-cadherin to induce EMT in lung cancer.	[48]
miRNA	TGF-β affects EMT by regulating miRNAs. This compound plays a significant role in tumor progression and can regulate the TGF-β pathway at multiple levels.	[50]
Integrin β3	TGF-β affects ↑ increased integrin β3 expression, which is associated with increased tumor cell invasiveness.	[52]
Lymphocytes T reg.	TGF-β affects the increase in levels of circulating T regulatory lymphocytes through the Cox-2/PGE2 pathway. Regulatory lymphocytes can inhibit the proliferation of CD4+ T cells, allowing tumor cells to evade the immune response.	[49]
PD-L1	The TGF-β1/Smad2 signaling pathway causes ↑ levels of PD-L1. PD-L1 can bind to PD-1, ultimately suppressing anti-tumor immunity in NSCLC.	[58]
Stromal reaction	TGF-β coordinates tumor stroma development and promotes angiogenesis, immune evasion, and extracellular matrix remodeling. The likely TGF-β-dependent stromal response is associated with a poor prognosis in resected lung adenocarcinoma.	[57]

**Table 3:** The importance of transforming growth factor β (TGF-β) in the progression and inhibition of colorectal cancer.

Factor/Process	Description	Source
TGFBR2	It transforms normal colon epithelial cells into malignant cells and, when combined with inflammation of the colon, causes invasive colon cancer.	[63]
Glycosylation	TGF-β can modulate the pattern of protein glycosylation in MSI-H CRC. Impaired TGFBR2 in this cell line causes ↑ an increase in the expression of specific glycosylation-related genes.	[63]
USF2/S100A8	TGF-β induces the upregulation of S100A8 and promotes the transcriptional activity of USF2, which facilitates EMT in which both factors are involved.	[65]
Signal route TGF-β	Eight TGF-β pathway proteins have been observed ↑ to be increased in tumor tissues; TGF-β signaling pathway inactivation increases tumor aggressiveness; Activation of TGF-β signaling prolongs OS; Activated TGF-β signaling prevents early-stage tumor progression.	[65,66]
TME contribution	TGF-β from stromal cells shapes growth, immunity, and interacts with genetic lesions.	[66]

**Table 4:** The role of transforming growth factor-β (TGF-β) in breast cancer progression.

Factor/Process	Description	Source
FBXO3	TGF-β1 stabilizes FBXO3, which in turn degrades ΔNp63α, thereby enhancing TGF-β signaling-induced EMT and tumor metastasis.	[23]
ΔNp63α	TGF-β1 causes a significant ↓ decrease in ΔNp63α levels, which inhibits metastasis in advanced cancers. Activation of TGF-β signaling promotes proteasomal degradation of ΔNp63α.	[23]
EGFR	TGF-β induces ↑ EGFR expression levels, whose signaling promotes breast cancer development.	[79]
NET	TGF-β activation causes cancer cells to undergo EMT, which correlates with NET-induced chemoresistance.	[80]
SOX4	TGF-β causes ↑ an increase in SOX4 expression, which induces resistance in cancer cells to cytotoxic T lymphocytes.	[81]

**Table 5:** Examples of therapies targeting the TGF-β pathway include antibodies, vaccines, and kinase inhibitors.

Antibody/Vaccine/ Inhibitor	Mechanism of Action	Type of Cancer	Study Phase	Results	Source
Fresolimumab (GC1008)	TGF-β1, β2, and β3 neutralizing antibody	Advanced melanoma, renal cell carcinoma	Phase I	The drug was well tolerated. 3/23 patients with melanoma achieved partial response (PR); Two patients with renal cell carcinoma achieved disease stabilization (SD).	[97]
264RAD	Monoclonal antibody blocking integrin αvβ6 (inhibition of TGF-β activation)	Breast cancer (HER2^+^, trastuzumab-resistant)	Preclinical phase	Inhibition of tumor growth and metastasis in in vivo models. More effective in combination with trastuzumab.	[98]
Lucanix (Belagenpumatucel-L)	Allogeneic TGF-β2 antisense gene vaccine	NSCLC	Phase III	Median OS: 20.3 months (vs. 17.8 months in the placebo group); Well tolerated and safe. Additional trials of combination therapy were recommended.	[99]
sTβRII-Fc and sBetaglycan-Fc	Soluble receptor-activated TGF-β neutralizers	Preclinical models of breast cancer (MDA-MB-231)	Preclinical phase	Inhibition of tumor growth and lung metastasis. Reduction in tumor cell motility. Induction of apoptosis.	[89]
Galunisertib (LY2157299)	Selective inhibitor of TGFBR1 kinase (ALK5); blocks TGF-β signaling	NSCLC, hepatocellular carcinoma	Phase I/II	NSCLC: ORR 24%, PFS 5.3 months, OS ~12 months; HCC: encouraging signals of efficacy and safety (in combination with sorafenib or as monotherapy).	[81]
Vactosertib (TEW-7197)	Selective inhibitor of TGFBR1/ALK5 kinase	Various advanced solid tumors	Phase I	Nontoxic and well tolerated. In the Phase I study, the overall response rate (ORR) was 21–32%, the disease control rate (DCR) was 47%, and the clinical benefit rate was approximately 69%.	[100]
LY3200882	Selective inhibitor of TGFBR1/ALK5 kinase	Pancreatic cancer, glioma	Phase I	Gliomas: PR in 3 of 4 patients. Pancreatic cancer: in combination with gemcine + nab-paclitaxel: 50% ORR and 75% DCR, a promising signal of efficacy at an early clinical stage.	[101]
